# Implications of Individual QT/RR Profiles—Part 2: Zero QTc/RR Correlations Do Not Prove QTc Correction Accuracy in Studies of QTc Changes

**DOI:** 10.1007/s40264-018-0735-2

**Published:** 2018-09-25

**Authors:** Marek Malik, Christine Garnett, Katerina Hnatkova, Jose Vicente, Lars Johannesen, Norman Stockbridge

**Affiliations:** 1 National Heart and Lung Institute, Imperial College, Dovehouse Street, London, SW3 6LY England UK; 2 Division of Cardiovascular and Renal Products, Office of New Drugs, Center for Drug Evaluation and Research, US Food and Drug Administration, Silver Spring, MD USA; 3 Division of Clinical Pharmacology I, Office of Clinical Pharmacology, Center for Drug Evaluation and Research, US Food and Drug Administration, Silver Spring, MD USA

## Abstract

**Introduction:**

In studies of drug-induced corrected QT (QTc) changes, fixed universal heart rate (HR) corrections (e.g., the Fridericia correction) are potentially misleading when assessing the effects of drugs that change HR. When data-specific corrections are designed, tests of their validity are needed. The proposed tests include zero correlations between QTc and corresponding RR values in the complete study data (pooling on-treatment and off-treatment interval measurements).

**Objective:**

To document that this approach is potentially highly misleading, a statistical modeling study was conducted based on the full profiles of QT/RR data of 523 healthy subjects—254 females, mean age 33.5 years.

**Methods:**

In each of the subjects, 50 baseline QT/RR readings were selected to model baseline data. In repeated experiments, groups of ten and 50 subjects were randomly selected and drug-induced HR increases between 0 and 25 beats per minute combined with QTc changes between − 20 and + 20 ms were modeled. In each experiment, subject-specific as well as population-specific HR corrections were designed so that the QTc interval data were uncorrelated to the corresponding RR interval data.

**Results:**

The simulation experiments showed that when zero correlations of QTc data with RR data are combined with more than trivial HR increases, the HR corrections are substantially biased and underestimate or fully eliminate any drug-induced QTc interval changes. This result is in full agreement with theoretical considerations of HR correction principles.

**Conclusions:**

The lack of correlation of QTc versus RR durations including on-treatment data does not prove any validity of HR corrections. Correlations of QTc versus RR in study data pooling on- and off-drug measurements should not be used to prove the appropriateness of HR corrections.

**Electronic supplementary material:**

The online version of this article (10.1007/s40264-018-0735-2) contains supplementary material, which is available to authorized users.

## Key Points

**Table:** 

In clinical studies of drug-induced corrected QT (QTc) changes, correlation coefficients between heart rate corrected QTc intervals and corresponding RR intervals pooled over all parts of the study have no valid meaning in justification of the heart rate correction methodology.
In the presence of combined drug-induced heart rate and QT interval changes, it appears problematic to assess the validity of QTc heart rate corrections based on the on-treatment data.

## Introduction

In studies of drug-induced corrected QT (QTc) changes, fixed universal heart rate corrections (e.g., the Fridericia correction) are potentially misleading when assessing the effects of drugs that change heart rate [1]. For such cases, subject-specific corrections have been proposed to avoid the possibility of inaccurate results. Nevertheless, the methods for forming the subject-specific corrections are frequently debated, and the appropriateness of some of the designs of these corrections may be questioned [2]. Consequently, tests of subject-specific corrections are needed.

As one of the possibilities of testing the validity of QT corrections designed for a specific QTc study, correlations between QTc intervals and the corresponding RR intervals are used. It is assumed that if all QTc data of a study (i.e., baseline, placebo, and active-treatment readings combined) are found uncorrelated to the corresponding RR intervals, the correction method is, in principle, validated, increasing the trust in the observed study results.

In this text, we show that this assumption is invalid and that the independency of QTc and RR intervals in the complete study data may be substantially misleading and may mask substantial drug-induced QTc changes. For this purpose, we have extended a statistical modeling study described in part 1 of “Implications of Individual QT/RR Profiles” [3].

## Theoretical Considerations

Before demonstrating the effects of achieving zero correlation between QTc and RR, the following theoretical considerations need to be presented.

**Fig. 1:**
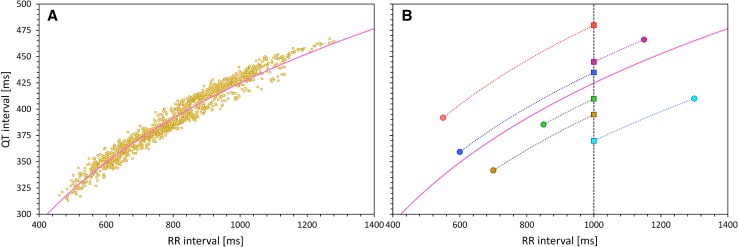
Schematic principles of heart rate correction. Panel **a** shows drug-free QT/RR data distribution in a healthy subject (small yellow marks) and their non-linear regression curvature (light violet line). Panel **b** shows the way in which the curvature is used for the purposes of heart rate correction. For different readings of QT and RR interval data (different colored circles), a line parallel to the correction curvature is used to shift the QT/RR readings to the line of RR = 1 s. QTc values (squares of corresponding colors) are obtained in this way. The mathematics of different heart rate corrections always works in this way albeit sometimes after the logarithmic (or some other) transformation of the scales of the QT and RR interval data. *QTc* corrected QT

As explained in detail in the guidance document by the Cardiac Safety Research Consortium [1], every heart rate correction is represented by a line or curvature passing through the plane of QT/RR data combinations. A QT interval duration measured at a certain RR interval (representing heart rate underlying the measurement) is corrected for heart rate by moving the QT/RR data point along the curvature of the heart rate correction until the RR value corresponds to the heart rate of 60 beats per minute (bpm) (i.e., RR of 1 s, which has historically, albeit arbitrarily, been selected as the rate at which QT durations are compared). This process is schematically shown in Fig. 1.

**Fig. 2:**
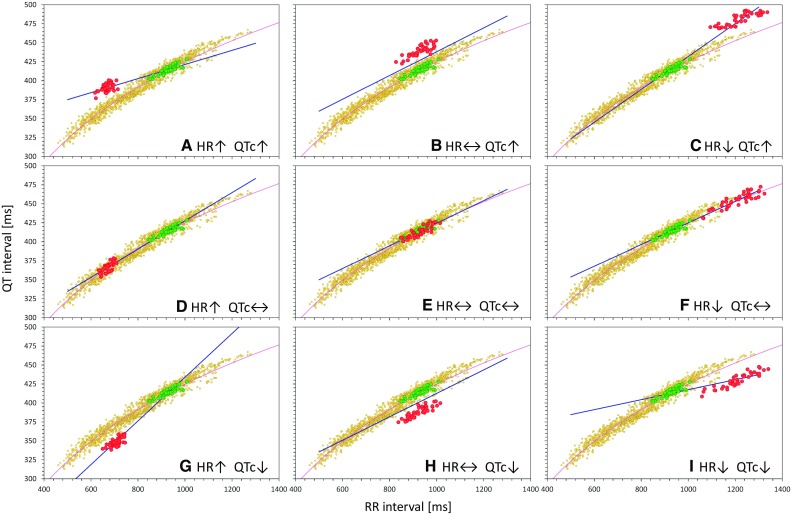
Theoretical cases of different combinations of HR and QTc interval changes. In each panel, the small yellow dots represent the full drug-free QT/RR data profile and the light violet line the curvature of their distribution. The green circles represent the baseline off-treatment data points, while the red circles represent the on-treatment data points. The blue lines show linear regression lines that, if used as the curvatures for HR correction, lead to the zero correlation between QTc and RR values for all the green and red readings combined. Panels in the top (**a**–**c**), middle (**d**–**f**), and bottom (**g**–**i**) row show cases when the investigated drug increases, does not change, and decreases the QTc interval, respectively. Panels in the left (**a**, **d**, and **g**), middle (**b**, **e**, and **h**) and right (**c**, **f**, and **i**) column show cases when the investigated drug increases, does not change, and decreases HR, respectively. Note that the blue regression lines are reasonably parallel with the full QT/RR curvatures (the violet lines) only in cases of no HR change when fixed HR corrections (e.g., Fridericia correction) can be fully relied on. *HR* heart rate, *QTc* corrected QT

**Fig. 3:**
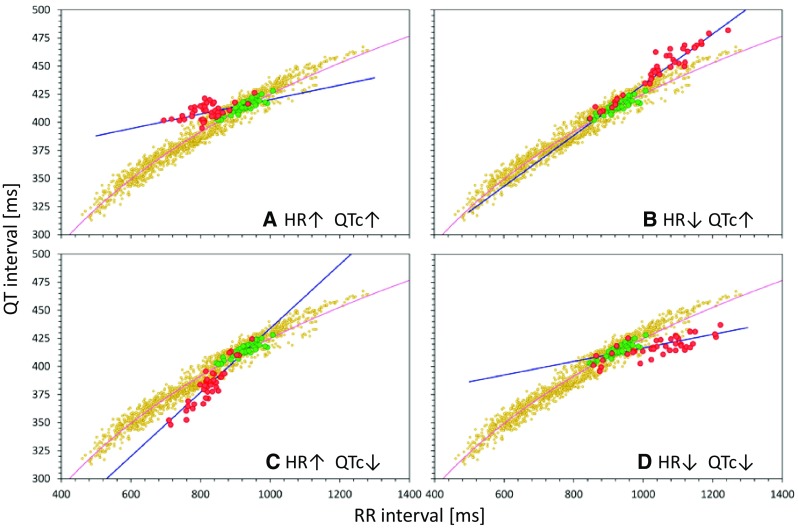
Theoretical cases of different combinations of gradual HR and gradual QTc interval changes. The layout of individual panels is the same as in Fig. 2, but instead of systematic HR and QT interval changes in all on-treatment data points, the panels of the figure show gradual changes between zero and maximum change. Individual panels correspond to the combination of HR increase + QT increase (panel **a**), HR decrease + QT increase (panel **b**), HR increase + QT decrease (panel **c**), and HR decrease + QT decrease (panel **d**). Compare with panels **a**, **c**, **g** and **i** of Fig. 2. *HR* heart rate, *QTc* corrected QT

This means that if a drug accelerates heart rate as well as prolongs QTc interval (such as the red case on the top in Fig. 1b), QTc intervals at shorter RR (on-treatment) are longer than QTc intervals at longer RR (baseline or placebo). When such QTc/RR pairs are pooled from all study data, the correlation between QTc and RR must be negative (see examples in Figs. 2a and 3a, as described further).

Similar considerations apply to other combinations of QT and heart rate changes. As shown in Fig. 2, nine combinations of increase/no change/decrease of QT and RR intervals may be considered. In each such combination, zero correlation between QTc and RR is only achieved if the curvature of the heart rate correction passes through the clouds of both on-treatment and off-treatment QT/RR data points. The slopes of such corrections (i.e., the direction along which the QT/RR point needs to be shifted to the level of RR = 1 s) can be very different from the valid heart rate correction that reflects the drug-free dependency of QT intervals on the underlying heart rate.

Figure 2 shows cases in which the heart rate and QT interval change between baseline (or placebo) and on-treatment data is relatively stable (e.g., after multiple drug doses that lead to almost constant plasma levels). Nevertheless, the same influence on the correction slopes also exists in situations when the heart rate and QT interval changes are gradual during study conduct, e.g., after a single drug dose when the changes gradually appear and subsequently subside. In such situations, the baseline/placebo data and the on-treatment data do not form separate “clouds” of data points (as seen in Fig. 2), but the on-treatment data are distributed between the no-effect and maximum-effect extremes. This is shown in Fig. 3, which demonstrates that in these cases, the slopes of corrections assuring zero correlation between QTc and RR are also influenced in practically the same way as was the case in Fig. 2.

**Fig. 4:**
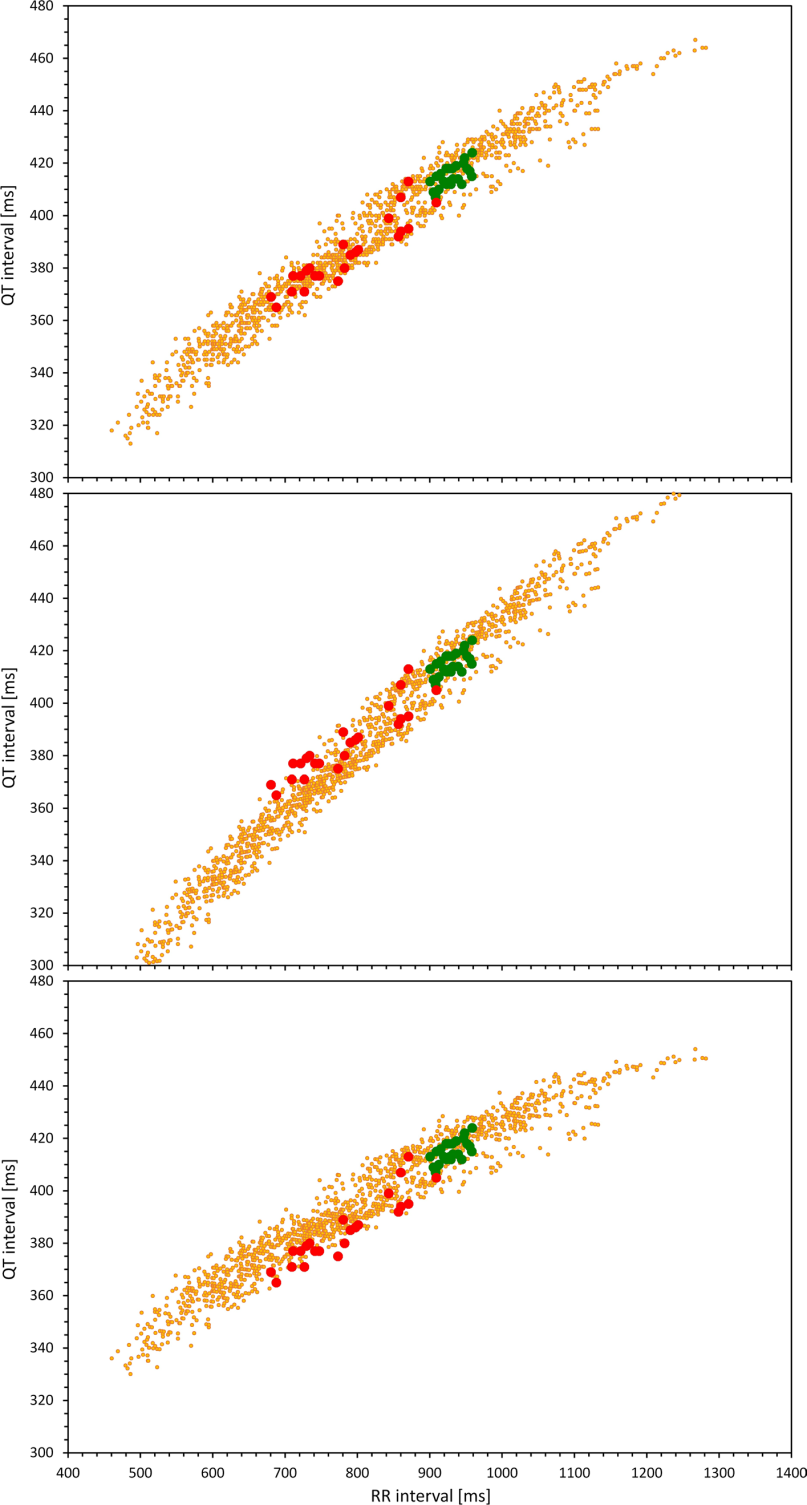
Theoretical cases showing the baseline QT/RR data over a restricted span of heart rates (green circles) and on-treatment data with gradual increases of heart rate and QT interval changes (red circles). In each panel, these data are superimposed on a full drug-free QT/RR profile over a wide range of heart rates (small yellow circles). Note that in all three panels, the green and red circles are exactly the same. Only the differences in the underlying full drug-free QT/RR profiles determine whether the drug only changes the heart rate but does not additionally change the QTc intervals (top panel—note that the red dots are within the drug-free QT/RR profile) or increases the QTc intervals (middle panel—note that with increasing heart rate acceleration, the red dots tend to be above the drug-free QT/RR profile) or decreases the QTc intervals (bottom panel—note that with increasing heart rate acceleration, the red dots tend to be below the drug-free QT/RR profile). *QTc* corrected QT

Finally, Fig. 4 demonstrates that if the drug-free baseline data are obtained over a narrow range of heart rates and if the investigated drug changes heart rate, it is impossible to interpret the on-treatment QT/RR data without knowing the full profile of the drug-uninfluenced QT/RR relationship over a broad range of heart rates. Note that the three panels of Fig. 4 show the same baseline and on-treatment data and that they differ only in the full QT/RR profiles. Hence, even when involving the underlying plasma levels of the drug [2] (again, the very same in the three panels of Fig. 4), no QTc/RR correlation restricted to the baseline and on-treatment data can distinguish between the cases when the drug increases and decreases the QTc intervals.

Hence, these theoretical considerations suggest that achieving zero correlation between QTc and RR interval data pooled from all parts of a study might be very inappropriate and might suppress the signal of drug-induced QTc changes. These considerations also show that the effect of enforcing zero correlations between all QTc and RR interval data is “unidirectional” in the sense that it might suppress drug-induced QTc changes, but it will not magnify them or produce erroneous QTc changes when none exist.

## Methods

To investigate the effects predicted by these theoretical considerations, we have extended the statistical modeling study described in part 1 of the series of simulation experiments [3].

In brief, we have used the data of the QT/RR relationship of 523 investigated healthy subjects—254 females, mean age 33.5 years [4]. In each subject, the description of the drug-free QT/RR profile was available in the form of non-linear regression QT = QTcI + (*δ*/*γ*)(RR^*γ*^ − 1), corresponding to the subject-specific correction formula QTcI = QT + (*δ*/*γ*)(1 − RR^*γ*^), where QTcI is the individually corrected QT interval, and individually optimized parameters *δ* and *γ* represent the slope and the curvature of the QT/RR relationship [5].

In each subject, we selected ten baseline data points (corresponding to 0, 1, 2, 3, 4, 5, 6, 8, 10, and 12 h after dosing), each represented by five replicates of QT and RR (hysteresis corrected [6]) interval measurements. The baseline data points were obtained while the study subjects maintained strict supine positions for at least 5 min before the QT measurements. The hysteresis correction utilized individual-specific exponential decay models, which provided weighted averages of RR intervals in 5 min preceding the QT interval measurement. Thus, these selected ten baseline data points were used to model the off-treatment data of standard QTc investigations.

As also described in more detail in part 1 [3], the non-linear regression description of the QT/RR relationship in each subject allowed us to simulate situations in which modeled on-treatment data differed from the selected baseline time points by a given heart rate and QTc difference. The same modeling formulas as described in part 1 were used, including the random inaccuracy coefficients.

### Statistical Modeling Experiments

Similar to the part 1 set of modeling experiments [3], two types of experiments were performed.

The experiments of the first type modeled situations of steady-state drug effects. In each of these experiments, we considered a randomly selected group of 50 subjects, and in each subject, on-treatment QT/RR data were considered, differing from the selected baseline time points by a programmed heart rate change and a programmed QTc interval change.

The experiments of the second type modeled situations in which a smaller group of subjects is investigated after a singular drug dose. The experiment assumed that the averaged plasma levels (and correspondingly the heart rate and QT interval effects) followed the modeled concentration profile as used in part 1 (see Fig. 1a of [3]) and that the baseline data points were distributed along the time axis in the same way as in the part 1 set of experiments. As before, each of these experiments considered a randomly selected group of ten subjects and each experiment modeled a certain heart rate change and QTc interval change at the maximum plasma concentration of the hypothetical drug.

That is, the experiments of the first type corresponded to the theoretical considerations shown in Fig. 2, while the experiments of the second type corresponded to Fig. 3. In addition, the experiments differed in the size of the modeled population.

### Heart Rate Correction

In each of the modeling experiments, heart rate corrections were optimized to remove the correlation between QTc and RR intervals over all baseline (the selected ten baseline data points) and on-treatment data pooled together. For this purpose, linear and log-linear corrections were used to achieve the zero correlations either in individual subjects or in the population of the experiment. Thus, in more detail, the following four corrections were applied:

- Subject-specific linear corrections in the form QTc = QT + *ω*(1 − RR), in which the parameter *ω* was optimized for each subject separately so that this subject’s data (in the given experiment) led to zero correlation between all QTc and all RR (both baseline and modeled on-treatment).
- Subject-specific log-linear corrections in the form QTc = QT/RR^*ɷ*^ in which the parameter *ɷ* was again optimized for each subject and for the given experiment separately so that the correlation between all QTc and all RR was zero in the given subject.
- Population-specific linear corrections in the form QTc = QT + *Ψ*(1 − RR), in which the coefficient *Ψ* was optimized for the given experiment so that the correlation of all the experiment data (both baseline and modeled on-treatment of all subjects in the experiment together) was zero. The same coefficient *Ψ* was applied to all subjects of the experiment.
- Population-specific log-linear corrections in the form QTc = QT/RR^*Φ*^, in which the coefficient *Φ* was again optimized and applied to all subjects of the experiment to achieve zero correlation between all QTc and RR intervals of all subjects of the experiment pooled together.

The zero correlation coefficients between QTc and RR data (either in each subject separately or in the complete experiment population) were obtained by optimizing the correction parameters by the golden cut algorithm [7]. (For the linear heart rate corrections, the result was the same as that obtained from simple linear regression calculations.)

In addition to these experimental heart rate corrections, subject-specific non-linear heart rate corrections derived from full drug-free QT/RR profiles [5] were also used.

### Organization of Experiments and Statistics

In both experiments of the first and second type, heart rate changes on modeled treatment were systematically changed between 0 and 25 bpm heart rate acceleration (note that Figs. 2 and 3 of the theoretical considerations show that the problem is symmetrical in terms of heart rate acceleration and deceleration). The QTc interval changes on modeled treatment were systematically changed between − 20 and + 20 ms.

In the experiments of the first type, the upper single-sided 95% confidence interval of QTc changes was calculated for each of the selected time points, and the resulting estimate of QTc changes was the maximum value of these upper confidence intervals over all selected time points. In the experiments of the second type, the on-treatment time point at the time of the maximum plasma concentration was used in each experiment subject, and in these time points, the upper single-sided 95% confidence interval of QTc changes was calculated. In both types of experiments, the upper confidence intervals were calculated from values in individual subjects assuming normal distribution.

For each combination of programmed heart rate and QT changes of modeled treatment, both types of experiments were repeated 50,000 times with different selection of study groups (i.e., groups of 50 or ten subjects for experiments of the first and second type, respectively). That is, in each experiment, the group of subjects was randomly selected [8], the modeled on-treatment QT/RR data points were calculated for each subject, and the heart rate correction formulas were optimized to achieve the zero correlations between the QTc and RR intervals as described in the previous section. Subsequently, these heart rate corrections were applied to the data and the ΔΔQTc values calculated. (As in the part 1 series of experiments, the uncertainty coefficients incorporated also the zero-centered differences between baseline and placebo. The model thus simulates ΔΔQTc values according to the standard definition [1]).

The ΔΔQTc results of the repeated experiments with the same modeled heart rate and QTc changes on treatment were sorted, and the 5th, 10th, 20th, 30th, 40th, 50th, 60th, 70th, 80th, 90th and 95th percentiles of the distribution were obtained. These data were graphically displayed.

## Results

As in the part 1 experiments, the intra-individual regression residuals of the QTcI curvilinear regression models of full subject-specific QT/RR profiles were 5.64 ± 1.12 ms.

**Fig. 5:**
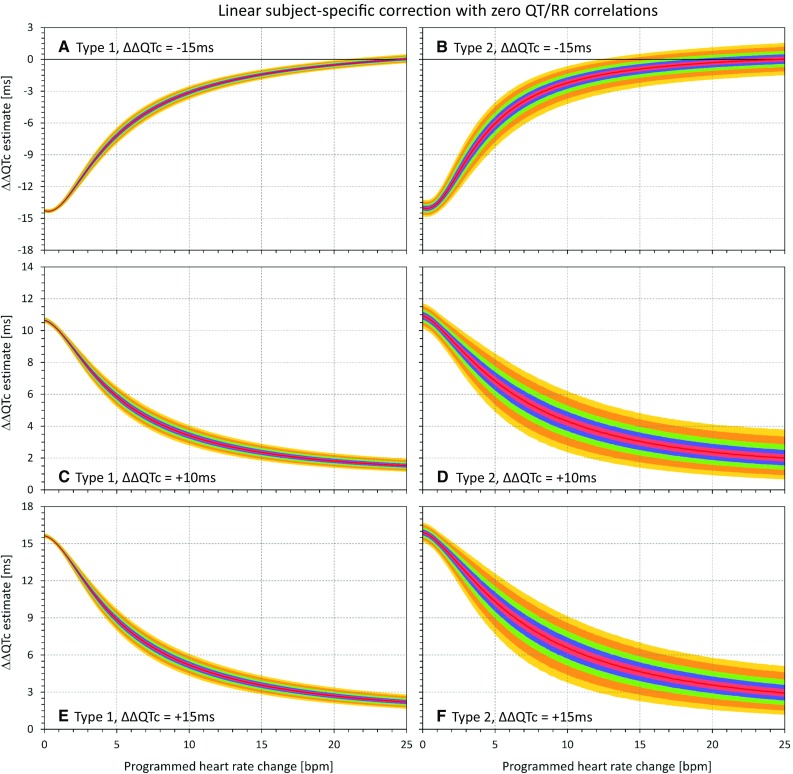
Results of the statistical modeling experiments that combined QTc shortening by 15 ms (panels **a**, **b**) and QTc prolongation by 10 ms (panels **c**, **d**) and by 15 ms (panels **e**, **f**) with different modeled heart rate increases. The results were obtained with subject-specific linear heart rate corrections that assured in each experiment and in each subject that all the QTc data (baseline and on-treatment) were uncorrelated to the corresponding RR interval data. Panels **a**, **c** and **e** show the results of the experiments of the first type (stable heart rate and QT changes in a larger population); panels **b**, **d**, and **f** show the results of the experiments of the second type (gradual heart rate and QT changes in a smaller population). In each panel, the red line shows the median value of the ΔΔQTc data distribution and the pink, blue, green, light brown, and yellow bands show the spread of the 40th–60th, 30th–70th, 20th–80th, 10th–90th, and 5th–95th percentiles, respectively. *QTc* corrected QT

**Fig. 6:**
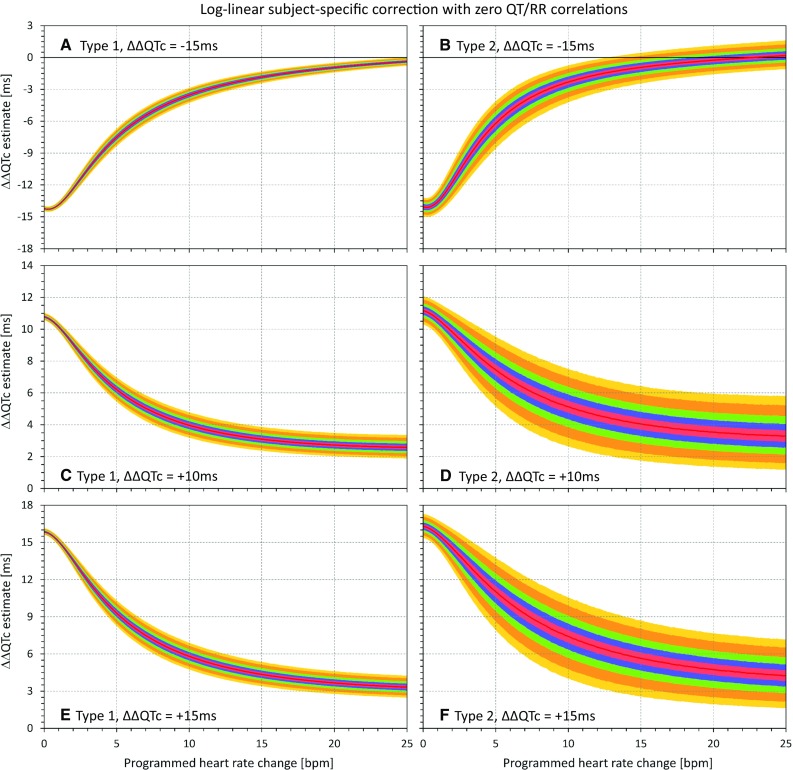
Results of the statistical modeling experiments that used subject-specific log-linear heart rate corrections that assured in each experiment and in each subject that all the QTc data (baseline and on-treatment) were uncorrelated to the corresponding RR interval data. The layout of the figure is the same as in Fig. 5. *QTc* corrected QT

**Fig. 7:**
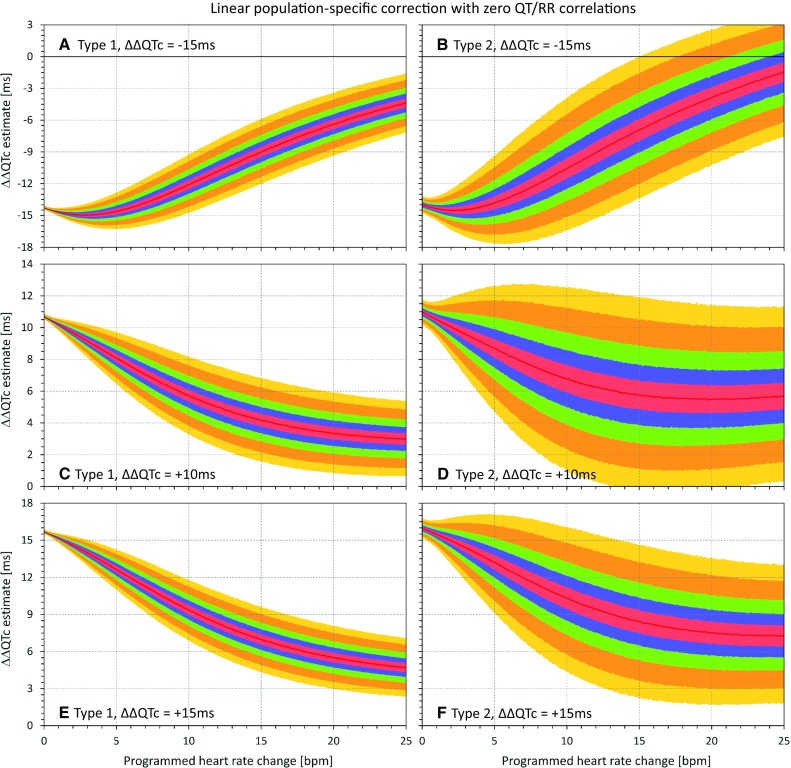
Results of the statistical modeling experiments that used population-specific linear heart rate corrections that assured in each experiment that all the QTc data (baseline and on-treatment pooled from all subjects) were uncorrelated to the corresponding RR interval data. The layout of the figure is the same as in Fig. 5. *QTc* corrected QT

**Fig. 8:**
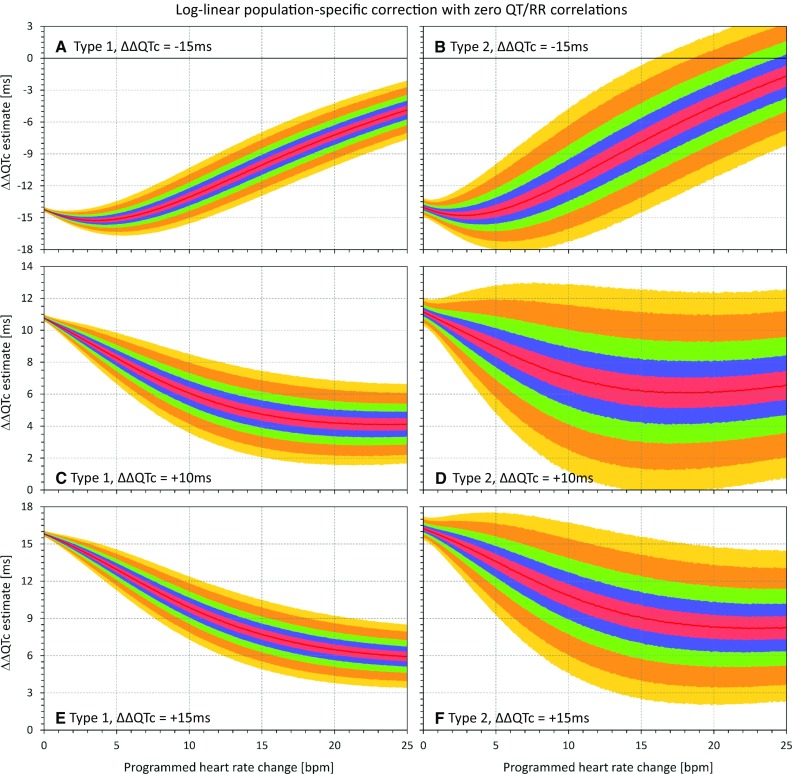
Results of the statistical modeling experiments that used population-specific log-linear heart rate corrections that assured in each experiment that all the QTc data (baseline and on-treatment pooled from all subjects) were uncorrelated to the corresponding RR interval data. The layout of the figure is the same as in Fig. 5. *QTc* corrected QT

Of the different combinations of modeled heart rate and QTc interval changes, Figs. 5, 6, 7, and 8 show the cases of QTc changes of − 15 ms, + 10 ms, and + 15 ms, combined with heart rate changes between 0 and 25 bpm in 0.1 bpm steps. Figures 5, 6, 7, and 8 correspond to the heart rate corrections (a), (b), (c) and (d), respectively.

In agreement with the theoretical considerations, the figures show that as heart rate changes increase, the ΔΔQTc values converge to zero, irrespective of the actual QTc interval changes modeled in the experiments.

Electronic supplementary material 1 shows that when using subject-specific heart rate corrections based on the full drug-free QT/RR profiles and uninfluenced by the modeled on-treatment data, the estimated ΔΔQTc values were independent of the modeled heart rate changes and corresponded closely to the modeled QTc changes (a slight overestimation of ΔΔQTc was caused by the introduction of random inaccuracies of modeled QTc). This is expected and corresponds to the set-up of the modeling experiments.

## Discussion

These statistical modeling experiments confirm the theoretical considerations. In the presence of drug-induced heart rate increases, QTc corrections that lead to zero correlation between QTc and RR are biased and tend to underestimate (or, in the presence of substantial heart rate changes, to fully eliminate) the true drug-induced QTc changes. Comparisons of different panels in Figs. 5, 6, 7, and 8 also show that the effects of eliminating QTc/RR correlations is “unidirectional” in the sense that in the presence of increased heart rates, the estimates of ΔΔQTc always converge to zero irrespective of whether the true programmed QTc changes are positive or negative. Apart from variability of ΔΔQTc estimates (as shown in the part 1 series of experiments [3]), no systematic magnification of true QTc changes occurs, but substantial true QTc changes (both increases and decreases) can be surpassed and masked by using heart rate corrections that eliminate the QTc/RR correlations. This is also in agreement with the theoretical considerations.

The larger spread of the ΔΔQTc results obtained with population-specific corrections in comparison to subject-specific corrections is contributed to by the problems of population corrections [3]. Nevertheless, the same effect of suppression of QTc changes in the presence of heart rate increase was also observed with these corrections.

In addition to the four heart correction possibilities described in Sect. 3.2, other correction methodologies can be designed and optimized to achieve zero correlations between all QT and RR data. For instance, a mixed effect model with fixed population correction effects and random subject correction effects can also be optimized to obtain the same zero correlations. While we have not included such models in our experiments, it appears logical that while the spread of their results would be between the spreads of the results that we obtained for individual and population corrections, the same trend towards substantial bias would exist in the presence of marked heart rate changes.

Both our theoretical considerations and the results of our experiments have implications for previous suggestions that considered pooling the off-drug and on-drug data together. If the investigated drug changes heart rate to a considerable degree so that the off-drug and on-drug heart rates are substantially distinct, valid drug-uninfluenced QT/RR profiles over sufficient spread of heart rates cannot, by definition, be obtained from a combination of off- and on-drug data. The concept of the so-called one-stage approach that was previously listed among other possibilities [1] and that aims at analyzing QT, RR, and drug data simultaneously appears to be invalidated by this series of experiments as well as by the theoretical considerations. Thus, the one-stage approach should be limited to situations in which the heart rate changes are unimportant [9], although in such situations, much simpler correction techniques (such as the Fridericia formula) appear to be sufficient and satisfactory [1].

The same criticism appears to apply to other methods that proposed relying on the QT/RR correlations within on-treatment data [10]. In the presence of combined drug-induced heart rate and QT interval changes, it is highly problematic attempting to assess the validity of heart rate corrections based on the on-treatment data.

Also, consistent with the theoretical considerations, the modeling experiments showed that the slopes of the heart rate correction curvatures that achieved zero QTc versus RR correlations were getting progressively less steep when progressive heart rate acceleration was combined with QTc increase. Similarly, the correction curvatures achieving zero QTc versus RR correlations were getting progressively steeper when progressive heart rate acceleration was combined with QTc decrease (data not shown).

In all the experiments, the estimated ΔΔQTc values corresponded to the modeled QTc changes only when the heart rate did not change on treatment or when the heart rate changes were minimal. This again corresponds to the theoretical predictions (see Fig. 2, middle column of panels) but more importantly, in such cases, specific design of heart rate corrections is not needed. With minimal heart rate changes on treatment, fixed heart rate corrections, such as the Fridericia formula, can safely be used [1].

Similar to part 1 experiments [3], the results presented in Figs. 5, 6, 7, and 8 are independent of the mathematical form of the curvilinear regression models that we used to describe the subject-specific drug-free QT/RR relationship over broad ranges of heart rates. Any other form of regression model that would fit the study data equally well would lead to equivalent results.

In the experiments, we have not considered QTc/RR correlation regressing QTc on both RR and the drug concentrations [2]. Nevertheless, as shown in Fig. 4 of the theoretical considerations, if such multiple correlations are based on a limited range of baseline RR values, no distinction can be made between drug-induced QTc prolongation or shortening (or no change). Hence, the QTc/RR correlations that involve drug concentrations suffer from the same shortcomings as demonstrated by our modeling experiments.

### Limitations

This series of statistical modeling experiments shares the limitations of the part 1 series [3], including the absence of more detailed QT/RR hysteresis considerations [6, 11], mixed sex population, restriction to healthy subjects, and absence of QRS complex abnormalities. Nevertheless, the theoretical considerations that we presented are equally applicable to QT/RR data obtained without any hysteresis correction, collected from patients rather than healthy subjects, etc. Hence, these experimental limitations do not influence the general validity of the concept that this study proves. Similar to the part 1 series, we have used by-time analysis estimating ΔΔQTc at the time of the maximum modeled drug concentration in each subject.

## Conclusions

In summary, this study and its theoretical basis show clearly that aiming at zero correlation between QTc and RR values by pooling baseline and on-treatment data does not prove validity of heart rate corrections used in study evaluation. When combined with more than trivial heart rate increases, such heart rate corrections may substantially underestimate drug-induced QTc changes.

For these reasons, the correlations between QTc and RR values over the complete study data collection should not be used. As discussed previously, full profiles of drug-free QT/RR data are needed in each study subject to design robust subject-specific corrections [1, 12]. Different tests validating the specifically designed heart rate corrections are needed, and in addition to previous proposals [13], new methodologies need to be developed for this purpose. In principle, it appears that with substantial drug-induced heart rate changes, it is problematic for the tests of correction validity to include the on-drug data.

## Electronic supplementary material

Below is the link to the electronic supplementary material.

Supplementary material 1 (PDF 693 kb)
