# Supplementary material for: Implications of Individual QT/RR Profiles—Part 2: Zero QTc/RR Correlations Do Not Prove QTc Correction Accuracy in Studies of QTc Changes
Source: Drug Saf. 2018 Sep 25;42(3):415–26. doi: 10.1007/s40264-018-0735-2 (PMC6426831; doi:10.1007/s40264-018-0735-2)
Supplement: Supplementary file 1 — Supplementary material 1 (PDF 693 kb) [file 40264_2018_735_MOESM1_ESM.pdf]

Article title: Implications of individual QT/RR profiles
Part 2: Zero QTc/RR correlations do not prove QTc correction accuracy in studies of QTc changes
Journal name: Drug Safety
Author names: Marek Malik (corresponding), Christine Garnett, Katerina Hnatkova, Jose Vicente, Lars Johannesen, Norman Stockbridge
Affiliation of corresponding author: National Heart and Lung Institute, Imperial College, Dovehouse Street, London SW3 6LY, England
Email of corresponding author: marek.malik@btinternet.com / marek.malik@imperial.ac.uk

Electronic Supplementary Material 1

Full QT/RR profile subject-specific correction with no requirement of QTc vs RR correlation

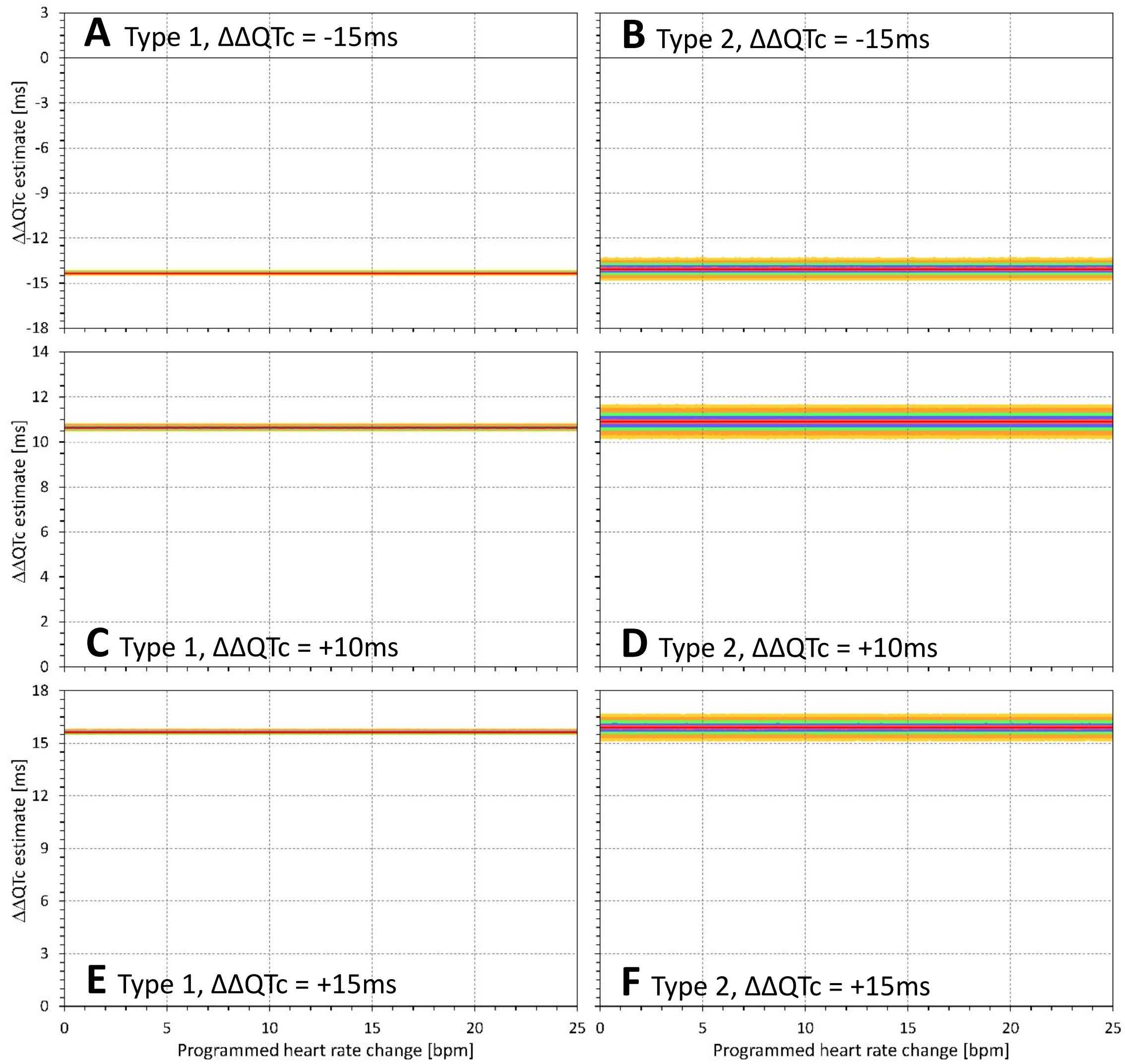

Results of the statistical modeling experiments that used curve-linear subject-specific heart rate corrections derived from the full drug-free QT/RR profile in each subject. The layout of the figure is the same as in Figure 4 of the main article. Compare with Figures 5, 6, 7, and 8 in which the heart rate corrections assured zero correlation between all QTc and RR intervals in each of the experiments.
